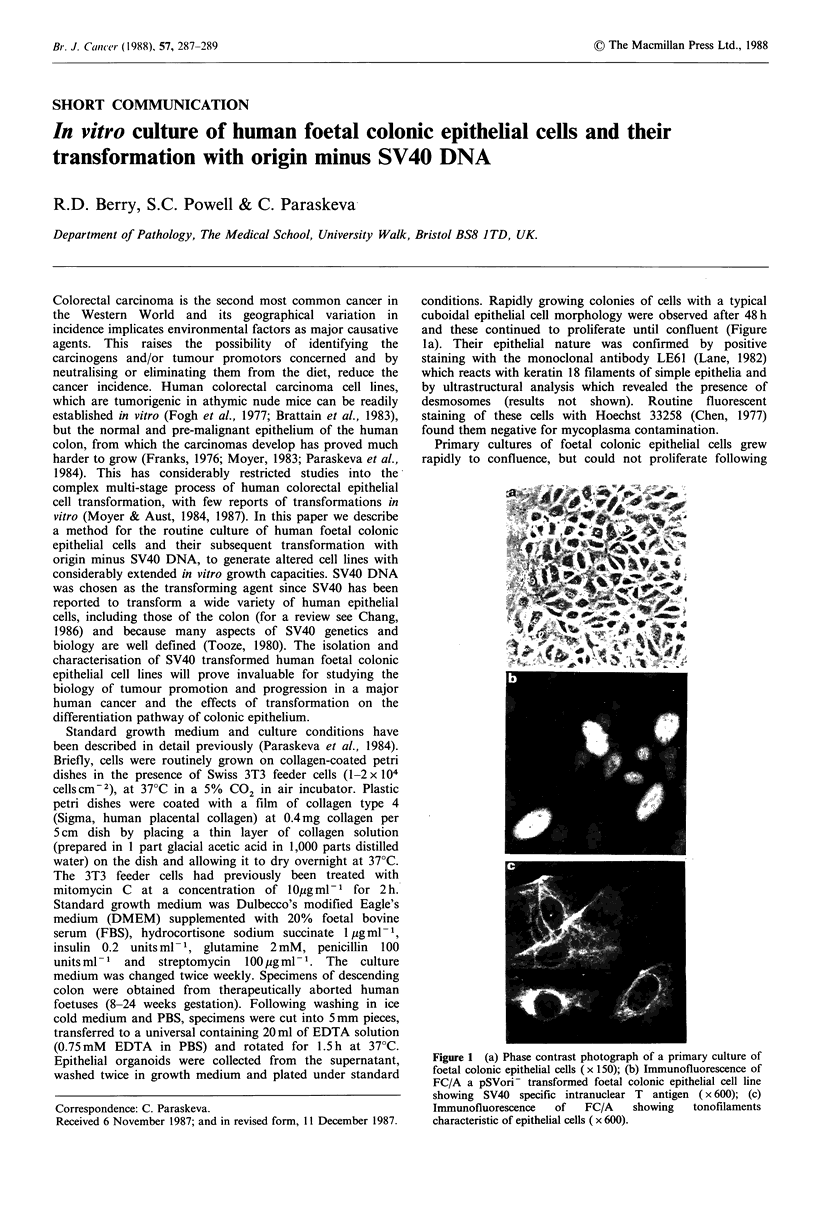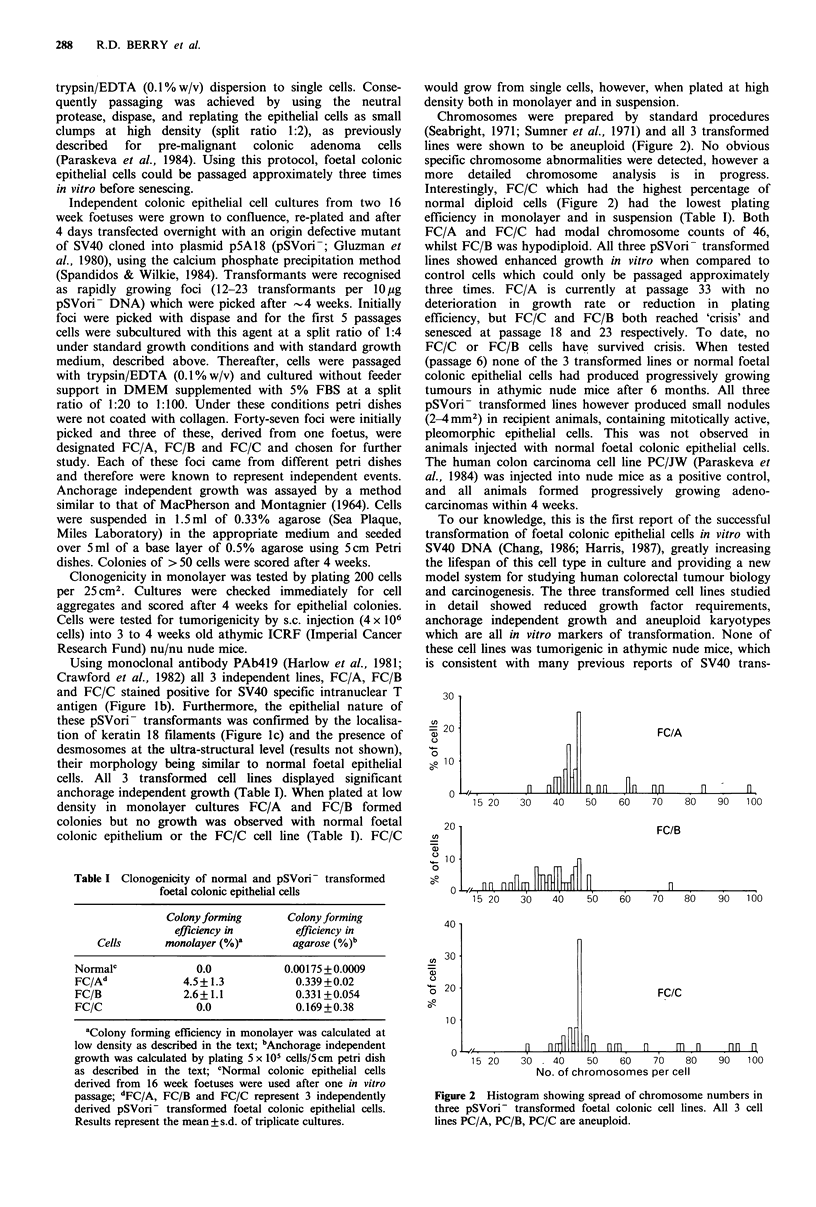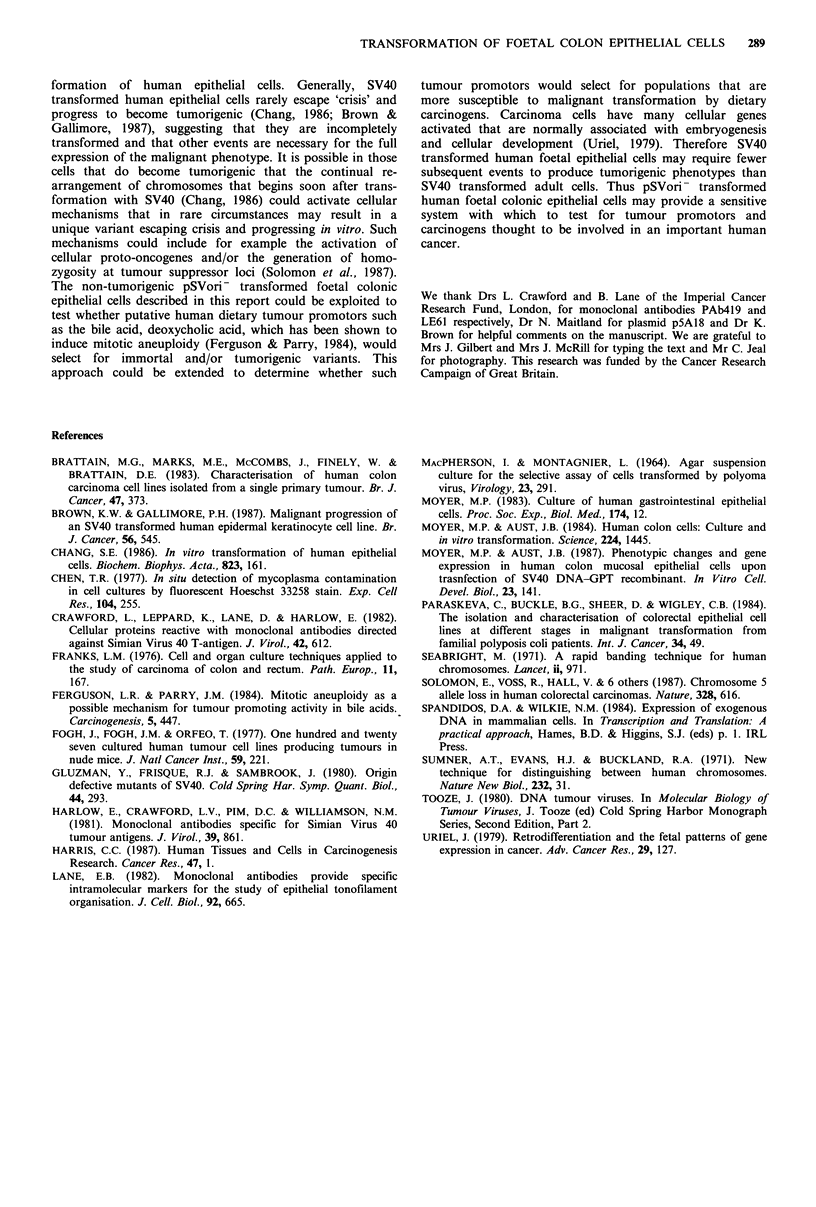# In vitro culture of human foetal colonic epithelial cells and their transformation with origin minus SV40 DNA.

**DOI:** 10.1038/bjc.1988.62

**Published:** 1988-03

**Authors:** R. D. Berry, S. C. Powell, C. Paraskeva

**Affiliations:** Department of Pathology, Medical School, Bristol, UK.

## Abstract

**Images:**


Br. J. Cancer (1988), 57, 287-289

© The Macmillan Press Ltd., 1988

SHORT COMMUNICATION

### *In vitro* culture of human foetal colonic epithelial cells and their transformation with origin minus SV40 DNA

R.D. Berry, S.C. Powell & C. Paraskeva

*Department of Pathology, The Medical School, University Walk, Bristol BS8 1TD, UK.*

Colorectal carcinoma is the second most common cancer in the Western World and its geographical variation in incidence implicates environmental factors as major causative agents. This raises the possibility of identifying the carcinogens and/or tumour promotors concerned and by neutralising or eliminating them from the diet, reduce the cancer incidence. Human colorectal carcinoma cell lines, which are tumorigenic in athymic nude mice can be readily established *in vitro* (Fogh *et al.*, 1977; Brattain *et al.*, 1983), but the normal and pre-malignant epithelium of the human colon, from which the carcinomas develop has proved much harder to grow (Franks, 1976; Moyer, 1983; Paraskeva *et al.*, 1984). This has considerably restricted studies into the complex multi-stage process of human colorectal epithelial cell transformation, with few reports of transformations *in vitro* (Moyer & Aust, 1984, 1987). In this paper we describe a method for the routine culture of human foetal colonic epithelial cells and their subsequent transformation with origin minus SV40 DNA, to generate altered cell lines with considerably extended *in vitro* growth capacities. SV40 DNA was chosen as the transforming agent since SV40 has been reported to transform a wide variety of human epithelial cells, including those of the colon (for a review see Chang, 1986) and because many aspects of SV40 genetics and biology are well defined (Tooze, 1980). The isolation and characterisation of SV40 transformed human foetal colonic epithelial cell lines will prove invaluable for studying the biology of tumour promotion and progression in a major human cancer and the effects of transformation on the differentiation pathway of colonic epithelium.

Standard growth medium and culture conditions have been described in detail previously (Paraskeva *et al.*, 1984). Briefly, cells were routinely grown on collagen-coated petri dishes in the presence of Swiss 3T3 feeder cells ($1\text{-}2\times10^4$ cells $cm^{-2}$), at 37°C in a 5% $CO_2$ in air incubator. Plastic petri dishes were coated with a film of collagen type 4 (Sigma, human placental collagen) at 0.4 mg collagen per 5 cm dish by placing a thin layer of collagen solution (prepared in 1 part glacial acetic acid in 1,000 parts distilled water) on the dish and allowing it to dry overnight at 37°C. The 3T3 feeder cells had previously been treated with mitomycin C at a concentration of $10 \mu g\,ml^{-1}$ for 2 h. Standard growth medium was Dulbecco's modified Eagle's medium (DMEM) supplemented with 20% foetal bovine serum (FBS), hydrocortisone sodium succinate $1 \mu g\,ml^{-1}$, insulin 0.2 units $ml^{-1}$, glutamine 2 mM, penicillin 100 units $ml^{-1}$ and streptomycin $100 \mu g\,ml^{-1}$. The culture medium was changed twice weekly. Specimens of descending colon were obtained from therapeutically aborted human foetuses (8–24 weeks gestation). Following washing in ice cold medium and PBS, specimens were cut into 5 mm pieces, transferred to a universal containing 20 ml of EDTA solution (0.75 mM EDTA in PBS) and rotated for 1.5 h at 37°C. Epithelial organoids were collected from the supernatant, washed twice in growth medium and plated under standard conditions. Rapidly growing colonies of cells with a typical cuboidal epithelial cell morphology were observed after 48 h and these continued to proliferate until confluent (Figure 1a). Their epithelial nature was confirmed by positive staining with the monoclonal antibody LE61 (Lane, 1982) which reacts with keratin 18 filaments of simple epithelia and by ultrastructural analysis which revealed the presence of desmosomes (results not shown). Routine fluorescent staining of these cells with Hoechst 33258 (Chen, 1977) found them negative for mycoplasma contamination.

Primary cultures of foetal colonic epithelial cells grew rapidly to confluence, but could not proliferate following

**Figure 1** (a) Phase contrast photograph of a primary culture of foetal colonic epithelial cells (×150); (b) Immunofluorescence of FC/A a pSVori⁻ transformed foetal colonic epithelial cell line showing SV40 specific intranuclear T antigen (×600); (c) Immunofluorescence of FC/A showing tonofilaments characteristic of epithelial cells (×600).

Correspondence: C. Paraskeva.
Received 6 November 1987; and in revised form, 11 December 1987.

trypsin/EDTA (0.1% w/v) dispersion to single cells. Consequently passaging was achieved by using the neutral protease, dispase, and replating the epithelial cells as small clumps at high density (split ratio 1:2), as previously described for pre-malignant colonic adenoma cells (Paraskeva *et al.*, 1984). Using this protocol, foetal colonic epithelial cells could be passaged approximately three times *in vitro* before senescing.

Independent colonic epithelial cell cultures from two 16 week foetuses were grown to confluence, re-plated and after 4 days transfected overnight with an origin defective mutant of SV40 cloned into plasmid p5A18 (pSVori$^-$; Gluzman *et al.*, 1980), using the calcium phosphate precipitation method (Spandidos & Wilkie, 1984). Transformants were recognised as rapidly growing foci (12–23 transformants per 10 $\mu$g pSVori$^-$ DNA) which were picked after ~4 weeks. Initially foci were picked with dispase and for the first 5 passages cells were subcultured with this agent at a split ratio of 1:4 under standard growth conditions and with standard growth medium, described above. Thereafter, cells were passaged with trypsin/EDTA (0.1% w/v) and cultured without feeder support in DMEM supplemented with 5% FBS at a split ratio of 1:20 to 1:100. Under these conditions petri dishes were not coated with collagen. Forty-seven foci were initially picked and three of these, derived from one foetus, were designated FC/A, FC/B and FC/C and chosen for further study. Each of these foci came from different petri dishes and therefore were known to represent independent events. Anchorage independent growth was assayed by a method similar to that of MacPherson and Montagnier (1964). Cells were suspended in 1.5 ml of 0.33% agarose (Sea Plaque, Miles Laboratory) in the appropriate medium and seeded over 5 ml of a base layer of 0.5% agarose using 5 cm Petri dishes. Colonies of >50 cells were scored after 4 weeks.

Clonogenicity in monolayer was tested by plating 200 cells per 25 cm$^2$. Cultures were checked immediately for cell aggregates and scored after 4 weeks for epithelial colonies. Cells were tested for tumorigenicity by s.c. injection ($4 \times 10^6$ cells) into 3 to 4 weeks old athymic ICRF (Imperial Cancer Research Fund) nu/nu nude mice.

Using monoclonal antibody PAb419 (Harlow *et al.*, 1981; Crawford *et al.*, 1982) all 3 independent lines, FC/A, FC/B and FC/C stained positive for SV40 specific intranuclear T antigen (Figure 1b). Furthermore, the epithelial nature of these pSVori$^-$ transformants was confirmed by the localisation of keratin 18 filaments (Figure 1c) and the presence of desmosomes at the ultra-structural level (results not shown), their morphology being similar to normal foetal epithelial cells. All 3 transformed cell lines displayed significant anchorage independent growth (Table I). When plated at low density in monolayer cultures FC/A and FC/B formed colonies but no growth was observed with normal foetal colonic epithelium or the FC/C cell line (Table I). FC/C would grow from single cells, however, when plated at high density both in monolayer and in suspension.

**Table I** Clonogenicity of normal and pSVori$^-$ transformed foetal colonic epithelial cells

| *Cells* | *Colony forming efficiency in monolayer* (%)[a] | *Colony forming efficiency in agarose* (%)[b] |
|---|---|---|
| Normal[c] | 0.0 | 0.00175±0.0009 |
| FC/A[d] | 4.5±1.3 | 0.339±0.02 |
| FC/B | 2.6±1.1 | 0.331±0.054 |
| FC/C | 0.0 | 0.169±0.38 |

[a]Colony forming efficiency in monolayer was calculated at low density as described in the text; [b]Anchorage independent growth was calculated by plating $5 \times 10^5$ cells/5 cm petri dish as described in the text; [c]Normal colonic epithelial cells derived from 16 week foetuses were used after one *in vitro* passage; [d]FC/A, FC/B and FC/C represent 3 independently derived pSVori$^-$ transformed foetal colonic epithelial cells. Results represent the mean±s.d. of triplicate cultures.

Chromosomes were prepared by standard procedures (Seabright, 1971; Sumner *et al.*, 1971) and all 3 transformed lines were shown to be aneuploid (Figure 2). No obvious specific chromosome abnormalities were detected, however a more detailed chromosome analysis is in progress. Interestingly, FC/C which had the highest percentage of normal diploid cells (Figure 2) had the lowest plating efficiency in monolayer and in suspension (Table I). Both FC/A and FC/C had modal chromosome counts of 46, whilst FC/B was hypodiploid. All three pSVori$^-$ transformed lines showed enhanced growth *in vitro* when compared to control cells which could only be passaged approximately three times. FC/A is currently at passage 33 with no deterioration in growth rate or reduction in plating efficiency, but FC/C and FC/B both reached 'crisis' and senesced at passage 18 and 23 respectively. To date, no FC/C or FC/B cells have survived crisis. When tested (passage 6) none of the 3 transformed lines or normal foetal colonic epithelial cells had produced progressively growing tumours in athymic nude mice after 6 months. All three pSVori$^-$ transformed lines however produced small nodules (2–4 mm$^2$) in recipient animals, containing mitotically active, pleomorphic epithelial cells. This was not observed in animals injected with normal foetal colonic epithelial cells. The human colon carcinoma cell line PC/JW (Paraskeva *et al.*, 1984) was injected into nude mice as a positive control, and all animals formed progressively growing adenocarcinomas within 4 weeks.

To our knowledge, this is the first report of the successful transformation of foetal colonic epithelial cells *in vitro* with SV40 DNA (Chang, 1986; Harris, 1987), greatly increasing the lifespan of this cell type in culture and providing a new model system for studying human colorectal tumour biology and carcinogenesis. The three transformed cell lines studied in detail showed reduced growth factor requirements, anchorage independent growth and aneuploid karyotypes which are all *in vitro* markers of transformation. None of these cell lines was tumorigenic in athymic nude mice, which is consistent with many previous reports of SV40 trans-

**Figure 2** Histogram showing spread of chromosome numbers in three pSVori$^-$ transformed foetal colonic cell lines. All 3 cell lines PC/A, PC/B, PC/C are aneuploid.

formation of human epithelial cells. Generally, SV40 transformed human epithelial cells rarely escape 'crisis' and progress to become tumorigenic (Chang, 1986; Brown & Gallimore, 1987), suggesting that they are incompletely transformed and that other events are necessary for the full expression of the malignant phenotype. It is possible in those cells that do become tumorigenic that the continual rearrangement of chromosomes that begins soon after transformation with SV40 (Chang, 1986) could activate cellular mechanisms that in rare circumstances may result in a unique variant escaping crisis and progressing *in vitro*. Such mechanisms could include for example the activation of cellular proto-oncogenes and/or the generation of homozygosity at tumour suppressor loci (Solomon *et al.*, 1987). The non-tumorigenic $pSVori^-$ transformed foetal colonic epithelial cells described in this report could be exploited to test whether putative human dietary tumour promotors such as the bile acid, deoxycholic acid, which has been shown to induce mitotic aneuploidy (Ferguson & Parry, 1984), would select for immortal and/or tumorigenic variants. This approach could be extended to determine whether such tumour promotors would select for populations that are more susceptible to malignant transformation by dietary carcinogens. Carcinoma cells have many cellular genes activated that are normally associated with embryogenesis and cellular development (Uriel, 1979). Therefore SV40 transformed human foetal epithelial cells may require fewer subsequent events to produce tumorigenic phenotypes than SV40 transformed adult cells. Thus $pSVori^-$ transformed human foetal colonic epithelial cells may provide a sensitive system with which to test for tumour promotors and carcinogens thought to be involved in an important human cancer.

We thank Drs L. Crawford and B. Lane of the Imperial Cancer Research Fund, London, for monoclonal antibodies PAb419 and LE61 respectively, Dr N. Maitland for plasmid p5A18 and Dr K. Brown for helpful comments on the manuscript. We are grateful to Mrs J. Gilbert and Mrs J. McRill for typing the text and Mr C. Jeal for photography. This research was funded by the Cancer Research Campaign of Great Britain.

### References

BRATTAIN, M.G., MARKS, M.E., McCOMBS, J., FINELY, W. & BRATTAIN, D.E. (1983). Characterisation of human colon carcinoma cell lines isolated from a single primary tumour. *Br. J. Cancer*, **47**, 373.

BROWN, K.W. & GALLIMORE, P.H. (1987). Malignant progression of an SV40 transformed human epidermal keratinocyte cell line. *Br. J. Cancer*, **56**, 545.

CHANG, S.E. (1986). *In vitro* transformation of human epithelial cells. *Biochem. Biophys. Acta.*, **823**, 161.

CHEN, T.R. (1977). *In situ* detection of mycoplasma contamination in cell cultures by fluorescent Hoeschst 33258 stain. *Exp. Cell Res.*, **104**, 255.

CRAWFORD, L., LEPPARD, K., LANE, D. & HARLOW, E. (1982). Cellular proteins reactive with monoclonal antibodies directed against Simian Virus 40 T-antigen. *J. Virol.*, **42**, 612.

FRANKS, L.M. (1976). Cell and organ culture techniques applied to the study of carcinoma of colon and rectum. *Path. Europ.*, **11**, 167.

FERGUSON, L.R. & PARRY, J.M. (1984). Mitotic aneuploidy as a possible mechanism for tumour promoting activity in bile acids. *Carcinogenesis*, **5**, 447.

FOGH, J., FOGH, J.M. & ORFEO, T. (1977). One hundred and twenty seven cultured human tumour cell lines producing tumours in nude mice. *J. Natl Cancer Inst.*, **59**, 221.

GLUZMAN, Y., FRISQUE, R.J. & SAMBROOK, J. (1980). Origin defective mutants of SV40. *Cold Spring Har. Symp. Quant. Biol.*, **44**, 293.

HARLOW, E., CRAWFORD, L.V., PIM, D.C. & WILLIAMSON, N.M. (1981). Monoclonal antibodies specific for Simian Virus 40 tumour antigens. *J. Virol.*, **39**, 861.

HARRIS, C.C. (1987). Human Tissues and Cells in Carcinogenesis Research. *Cancer Res.*, **47**, 1.

LANE, E.B. (1982). Monoclonal antibodies provide specific intramolecular markers for the study of epithelial tonofilament organisation. *J. Cell. Biol.*, **92**, 665.

MacPHERSON, I. & MONTAGNIER, L. (1964). Agar suspension culture for the selective assay of cells transformed by polyoma virus, *Virology*, **23**, 291.

MOYER, M.P. (1983). Culture of human gastrointestinal epithelial cells. *Proc. Soc. Exp., Biol. Med.*, **174**, 12.

MOYER, M.P. & AUST, J.B. (1984). Human colon cells: Culture and *in vitro* transformation. *Science*, **224**, 1445.

MOYER, M.P. & AUST, J.B. (1987). Phenotypic changes and gene expression in human colon mucosal epithelial cells upon trasnfection of SV40 DNA-GPT recombinant. *In Vitro Cell. Devel. Biol.*, **23**, 141.

PARASKEVA, C., BUCKLE, B.G., SHEER, D. & WIGLEY, C.B. (1984). The isolation and characterisation of colorectal epithelial cell lines at different stages in malignant transformation from familial polyposis coli patients. *Int. J. Cancer*, **34**, 49.

SEABRIGHT, M. (1971). A rapid banding technique for human chromosomes. *Lancet*, **ii**, 971.

SOLOMON, E., VOSS, R., HALL, V. & 6 others (1987). Chromosome 5 allele loss in human colorectal carcinomas. *Nature*, **328**, 616.

SPANDIDOS, D.A. & WILKIE, N.M. (1984). Expression of exogenous DNA in mammalian cells. In *Transcription and Translation: A practical approach*, Hames, B.D. & Higgins, S.J. (eds) p. 1. IRL Press.

SUMNER, A.T., EVANS, H.J. & BUCKLAND, R.A. (1971). New technique for distinguishing between human chromosomes. *Nature New Biol.*, **232**, 31.

TOOZE, J. (1980). DNA tumour viruses. In *Molecular Biology of Tumour Viruses*, J. Tooze (ed) Cold Spring Harbor Monograph Series, Second Edition, Part 2.

URIEL, J. (1979). Retrodifferentiation and the fetal patterns of gene expression in cancer. *Adv. Cancer Res.*, **29**, 127.